# Circulating miRNAs are generic and versatile therapeutic monitoring biomarkers in muscular dystrophies

**DOI:** 10.1038/srep28097

**Published:** 2016-06-21

**Authors:** David Israeli, Jérôme Poupiot, Fatima Amor, Karine Charton, William Lostal, Laurence Jeanson-Leh, Isabelle Richard

**Affiliations:** 1 INSERM, U951, INTEGRARE research unit, Evry, F-91002, France; 2 Généthon, Evry, F-91002, France

## Abstract

The development of medical approaches requires preclinical and clinical trials for assessment of therapeutic efficacy. Such evaluation entails the use of biomarkers, which provide information on the response to the therapeutic intervention. One newly-proposed class of biomarkers is the microRNA (miRNA) molecules. In muscular dystrophies (MD), the dysregulation of miRNAs was initially observed in muscle biopsy and later extended to plasma samples, suggesting that they may be of interest as biomarkers. First, we demonstrated that dystromiRs dysregulation occurs in MD with either preserved or disrupted expression of the dystrophin-associated glycoprotein complex, supporting the utilization of dystromiRs as generic biomarkers in MD. Then, we aimed at evaluation of the capacity of miRNAs as monitoring biomarkers for experimental therapeutic approach in MD. To this end, we took advantage of our previously characterized gene therapy approach in a mouse model for α-sarcoglycanopathy. We identified a dose-response correlation between the expression of miRNAs on both muscle tissue and blood serum and the therapeutic benefit as evaluated by a set of new and classically-used evaluation methods. This study supports the utility of profiling circulating miRNAs for the evaluation of therapeutic outcome in medical approaches for MD.

Significant progresses have been achieved in recent years in the development of therapeutic strategies for muscular dystrophies (MD)^1^,^2^,^3^. Most remarkable is that several approaches in Duchenne muscular dystrophy (DMD)^4^ that include the viral-mediated delivery of minidystrophin^5^, antisense oligonucleotide-mediated exon-skipping (for a recent review^6^) and the use of small-molecules for stop codon read-through or for the upregulation of utrophin expression^7^ have now reached the clinics. Viral-mediated delivery of the deficient genes have also been evaluated in clinical trials for other MD, namely limb girdle muscular dystrophies (LGMDs) 2C and 2D, which are caused by deficiencies in γ-sarcoglycan (*SGCG*) and α-sarcoglycan (*SGCA*), respectively^8^,^9^. These early translational studies in MD are being followed by a growing number of ongoing clinical trials^10^.

The choice of appropriate monitoring biomarker(s) to evaluate the efficacy of experimental therapy is particularly crucial in the DMD disease. Indeed, whereas recent development of therapeutic strategies has been extremely rapid, the choice of primary and secondary endpoints has been lagging behind^11^,^12^. The utility of quantification of the dystrophin itself, as a biomarker, is still under debate. Dystrophin level varies between muscle and biopsies, its quantification is technically uncertain, and its correlation to patients’ overall clinical improvement is under question^13^. In preclinical animal studies, it is relatively easy to obtain muscle biopsies which facilitate molecular characterization of the therapeutic progress. This is not the case in human trials, where minimally invasive monitoring methods are necessary. Currently such noninvasive methods include the evaluation of patients’ muscles’ physical capacity^14^,^15^, MRI based functional assessments of cardiac and skeletal muscles^16^,^17^,^18^, and quantification of circulating biomarkers. The most commonly used circulating biomarker for MD is serum muscle creatine kinase (mCK), which leaks into the blood stream upon muscle damage. However, mCK demonstrates variations due to physical activity, muscle injury, cramping, toxic agents or age^19^, and thus is of limited utility for disease assessment. Other dysregulated serum proteins in DMD disease, the muscle metalloproteinase-9 (MMP-9)^20^ and myomesin-3^21^, are under investigation as candidate biomarkers. Another class of circulating molecules that can potentially be used as monitoring biomarkers is the microRNAs (miRNAs). The use of miRNAs for diagnostic purposes in MD was suggested in 2007 by Eisenberg *et al*.^22^, who demonstrated distinct miRNA profiles in muscle biopsies of 10 primary muscular disorders. Concomitantly, it was discovered that miRNA molecules are stably expressed in body liquids and might potentially be used as disease biomarkers^23^,^24^. Subsequently, we and others have assessed the expression patterns of blood serum and plasma miRNAs and identified their dysregulation in DMD animal models and patients^25^,^26^,^27^,^28^,^29^,^30^,^31^,^32^, supporting thus the capacity of circulating miRNAs as biomarkers in DMD.

In this study, we investigated the utility of circulating miRNAs that are frequently dysregulated in distinct MDs for therapeutic monitoring. In the first part of this study, we evaluated the dysregulation of these miRNAs on two categories of MDs, the first with preserved expression of the dystrophin-associated glycoprotein complex (DAPC), and the second with disrupted DAPC structure. Evaluation of biomarker for therapeutic monitoring requires investigation of correlation between its expression or activity and the therapeutic benefit. To our knowledge, no published investigations have attempted at the evaluation of correlative relations between therapeutic benefit and serum miRNAs in a therapeutic set-up in MD. Therefore, in the second part of this study, we investigated the possibility to use circulating miRNAs as surrogate biomarkers in a mouse gene therapy model. This part of the investigation was carried out in the LGMD2D KO*-Sgca* mouse, taking advantage of a gene therapy approach that has been developed in our laboratory^33^.

For these purposes, we evaluated the muscle and serum expression of three categories of miRNAs (Table 1). The first category included the so-called dystromiRs, miR-1, miR-133 and miR-206, which are muscle enriched miRNAs dysregulated in the serum in DMD animal models and human patients^25^,^26^,^27^,^30^,^31^,^32^. The second category included miR-149-5p, miR-193b and miR-378a-3p, that were shown to be dysregulated in the serum in three distinct mouse models of MD^25^. The third miRNA category included miR-21, miR-31 and miR-142-3p which were reported to be dysregulated in muscle biopsies in mouse models for MD in relation to tissue fibrosis^34^, proliferation of regenerating myoblasts^35^, and inflammatory infiltration^36^, respectively. We are reporting here the dysregulation of all studied miRNAs in sera and muscles of dystrophic mouse models and the normalization of miRNAs expression upon treatment in a gene-therapy mouse model for MD.

## Results

### DystromiRs are dysregulated in dystrophic models in the serum and the muscle even when the DAPC is not disrupted

To investigate the relationship between the level of miRNA and the dystrophic level, a collection of 5 mouse models for MD, listed in Table 2, were analyzed at the ages of 1 and 6 months in comparison with their age and genetic-background matched C57Bl/6 healthy controls. The KO-*Sgca* mice were used as model for LGMD2D (α-sarcoglycanopathy)^37^, KO-*Sgcg* mice for LGMD2C (γ-sarcoglycanopathy)^38^, and *mdx*4cv mice for DMD^39^. They result from mutations in the α-sarcoglycan (*Sgca*), γ-sarcoglycan (*Sgcg*) and dystrophin (*Dmd*) genes, respectively, which all encode proteins of the DAPC, which connects the cytoskeleton of myofibers to the surrounding extracellular matrix through the cell membrane. The KO-*Dysferlin* mouse, was used as a model for LGMD2B^40^ and the KO-*Capn3* mouse was used as model for LGMD2A^41^. They result from mutations in the *Dysf* gene and the *Capn-3* genes, respectively. In these MD, the expression and cellular localization of the DAPC is preserved. Three of the studied strains, the KO-*Sgca*, the KO-*Sgcg* and the *mdx*, are models for myopathies that are thus characterized by disrupted expression of the DAPC and, by robust muscle regenerative process at least in young mice (For references, see Table 2). These three myopathies are designated here as the high-regenerating models (HRM). The two other strains, the KO-*Dysf* and the KO-*Capn3*, are characterized by preserved DAPC and a moderate muscle regeneration response and are designated here as the low-regenerating models (LRM) (Table 2).

**Figure 1:**
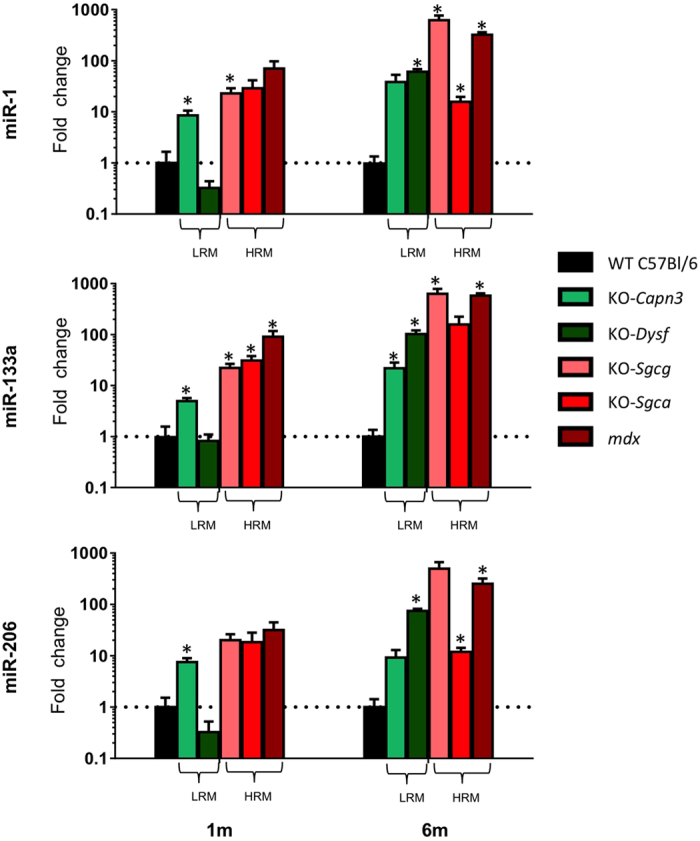
Quantification of serum miRNAs in mouse models for muscular dystrophies. Serum samples (n = 4) were collected from 1 (left) and 6 (right) month-old mice models for the indicated pathologies. MicroRNA expressions, normalized to miR-16, are expressed as Fold change (FC) relative to the control C57Bl/6 mouse. Significant differences (p < 0.05) are marked by*. LRM and HRM indicated low and high regenerative myopathies, respectively. All FC and p values are shown in Supplemental Table ST1.

First, expression levels of the dystromiRs, miR-1, miR-133a and miR-206 were quantified by RT-qPCR in the serum of the 5 models. With the exception of the KO-*Dysf* at the age of 1 month, the dystromiRs were upregulated in the serum of all disease models at both 1 and 6 months of age. DystromiR serum upregulation was generally higher in the HRM than in the LRM (with the exception of KO-*Sgca* at 6 month-old), and higher in 6 month-old than in 1 month-old mice (Fig. 1 and Supplemental Table ST1). Beyond the confirmation of the upregulation of the serum dystromiRs in all three HRM^25^, these data support their upregulation in the serum of the mouse models for the LRM as well, though to a lower level.

Expression of miRNA was also studied in the gastrocnemius (Ga) muscle in the same disease models and at the same ages. In addition to the dystromiRs, we quantified the muscle expression of three other miRNAs, miR-21, miR-31 and miR-142-3p, known to participate in three processes hallmark of MD. Indeed, the expression of miR-31 and miR-21 were shown to be upregulated in dystrophic muscle, in correlation with the formation of regenerating myoblasts for miR-31^35^ and the cellular fibrotic process for miR-21^34^,^42^. The miR-142-3P is highly expressing in monocytes^43^ and lymphocytes^44^, suggesting its enriched expression in muscle-invading inflammatory cells in MD (for a recent review see^45^).

**Figure 2:**
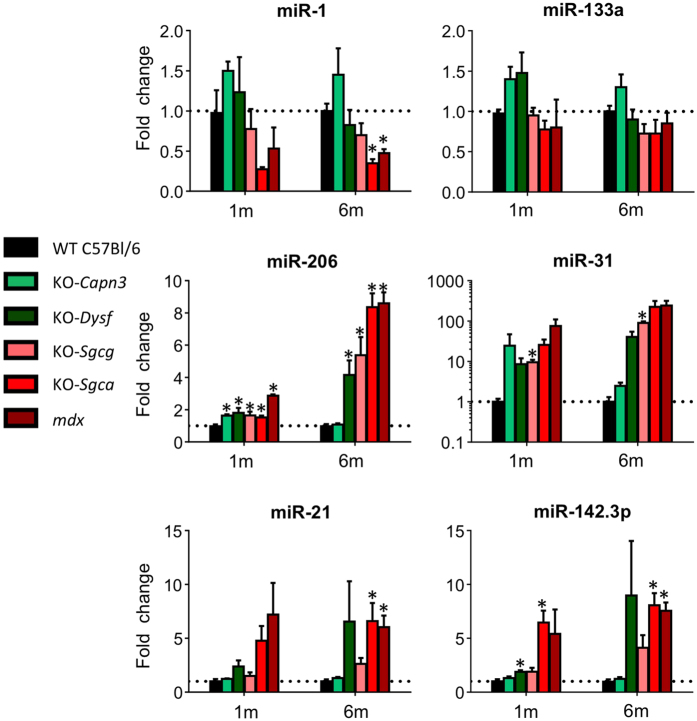
Quantification of muscle miRNAs in mouse models for muscular dystrophies. Biopsies of the gastrocnemius (Ga) muscle (n = 4) were collected from mice models for the indicated pathologies at the ages of 1 (left) and 6 (right) months. MicroRNA expressions, normalized to miR-93, are expressed as FC relative to the control C57Bl/6 mouse. Significant differences (p < 0.05) relative to the healthy control C57Bl/6 (WT-PBS) are marked by*. All FC and p values are shown in Supplemental Table ST2.

Globally, muscle miRNA dysregulation, comprising both up and down-regulation, was higher in the 6 month- than in the 1 month-old mice. In contrast to the sera, in muscle biopsies, miR-1 and miR-133a expressions were slightly lower than controls on the HRM, with statistical significance only for miR-1 in 6 month-old KO-*Sgca* and *mdx* models (Fig. 2 and Supplemental Table ST2). Marked upregulation in all except 6 month-old *Capn3* disease models was observed for miR-206, and also for miR-31 but less significantly (Fig. 2 and Supplemental Table ST2). Mir-21 and miR-142-3p were both upregulated, slightly in the LRM and to higher levels in the HRM. We established that the increase of miR-142-3p was contributed mainly by the infiltrating inflammatory cells within the dystrophic muscle since miR-142-3p expression was enriched in CD45 positive mono-nucleated cells derived from the muscle of the HRM KO-Sgca (Supplemental Figure SF1).

Taken collectively, we confirmed the dysregulation of dystromiRs in the serum and muscle of HRM, with disrupted DAPC, but also identified their dysregulation, though to a lesser extent, in the LRM, with preserved DAPC expression. Thus, the data support that dystromiRs dysregulation in MD occurs at least partially independently of DAPC-related pathophysiological mechanisms. Additionally, we confirmed inverse dysregulation in MD of miR-1 and miR-133a, between the muscle and the serum, rising in the serum and falling in the muscle.

### MiRNAs expression is normalized in the serum and muscle of a dystrophic mouse model treated by gene transfer

The KO-*Sgca* mouse model was used for the assessment of miRNAs as monitoring biomarkers in a preclinical gene transfer study. Five week-old KO-*Sgca* mice (n = 5) were intravenously injected with a recombinant adeno-associated virus (rAAV9) expressing the human α-sarcoglycan cDNA (SGCA) under the control of the desmin promoter. Mice were injected with escalating doses of the recombinant vector [4 × 10^12^, 2 × 10^13^ and 4 × 10^13^ viral genome (vg/kg)]. PBS injected Sgca–null and C57Bl/6 were used as negative and positive controls, respectively.

**Figure 3:**
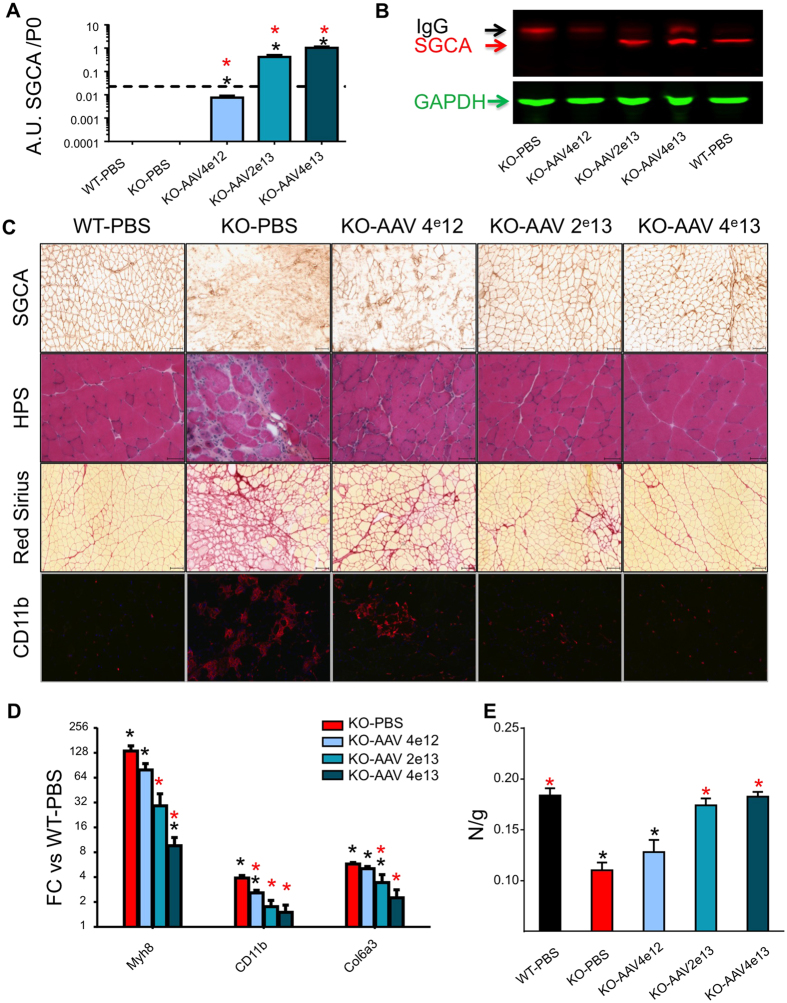
Gene delivery characterization and assessment in a mouse model for the LGMD2D (α-sarcoglycanopathy) muscular dystrophy. (**A**) Transgene mRNA expression in the gastrocnemius muscle. Expression of the human α-sarcoglycan is in arbitrary units (AU) relative to the endogenous P0 mRNA. Red and black asterisks indicate the significant difference of comparison respectively to untreated KO-*Sgca* (KO-PBS) and to healthy WT control mouse. (**B**) Western blot analysis of α-sarcoglycan expression in the Ga muscle of WT and KO mice injected with PBS or the AAV vector. The SGCA protein is indicated by the red arrow. The black arrow corresponds to endogenous immunoglobulins (IgG) and the green arrow indicates the GAPDH used as a normalizer. **(C**) Histological characterization of transversal sections of the gastrocnemius muscle in treated mice, with (from top to bottom) immuno-detection of α-sarcoglycan (SGCA), hematoxylin phloxine saffron (HPS), Red Sirius stainings, and CD11b immune-detection. (**D**) mRNA expression in the gastrocnemius of neonatal Myosin heavy chain 8 (Myh8), a marker of muscle regeneration^47^; CD11b, a marker of inflammatory infiltration, and collagen 6 isoform a3 (Col6a3), a marker of muscle fibrosis^48^. Expression levels are in arbitrary unit relative to the control C57Bl/6 mouse (WT-PBS). Red and black asterisks designate significant differences compared to untreated KO-*Sgca* (KO-PBS) and to healthy WT control (WT-PBS) mice, respectively. FC and p values are in Supplemental Table ST3. **(E)** Escape test. Assessment of muscle function by measurement of the pulling force generated by the mouse, expressed in Newton per body weight (N/g). Red and black asterisks indicate the significant difference compared to untreated KO-*Sgca* (KO-PBS) and to healthy WT control mouse (WT-PBS), respectively.

At the end-point of the experiment, the transgene expression and localization were monitored by RT-qPCR, Western blot and immunohistochemistry, indicating increased transgene mRNA and protein expressions in direct correlation to the injected level of the recombinant vector (Fig. 3A–C). A positive response was observed in the treated animals with improvement of the overall tissue architecture, as seen in the hematoxylin phloxine saffron (HPS) stained sections, a reduction of fibrosis and inflammatory infiltration as evaluated by Sirius Red and CD11b staining, respectively (Fig. 3C). Quantitative evaluation of this response was studied by RT-qPCR expression measurement of a set of indicator genes in MD. Neonatal myosin heavy chain 8 (Myh8) is a marker of muscle regeneration^46^,^47^. CD11b (shown qualitatively at the protein level in Fig. 3C) is a marker of muscle inflammatory infiltration, and collagen 6 isoform Col6a is a marker of fibrotic tissue in MD^48^. Expression of these indicators, highly upregulated in untreated KO-Sgca mice, were gradually reduced upon treatment, in correlation to the viral dose (Fig. 3D). Similarly, a viral vector dose-dependent reduction in mCK activity was detected in the treated mice in a longitudinal study (Supplemental Figure SF2), and the abnormal increased muscle weight, a pathological characteristic of this mouse model^33^, was gradually reduced (Supplemental Figure SF3). Lastly, the “escape” test, consisting of the measurement of the escaping force that is generated by the mouse, revealed a dose-dependent increase of muscle force in the treated mice (Fig. 3E), up to a level similar to the control healthy mice for the intermediate and high-doses treated KO-*Sgca* mouse.

Collectively and consistently with our previous study^33^, these results indicated an efficient transgene expression and a recovery of muscle architecture and function in the gene-transfer treated mice.

**Figure 4:**
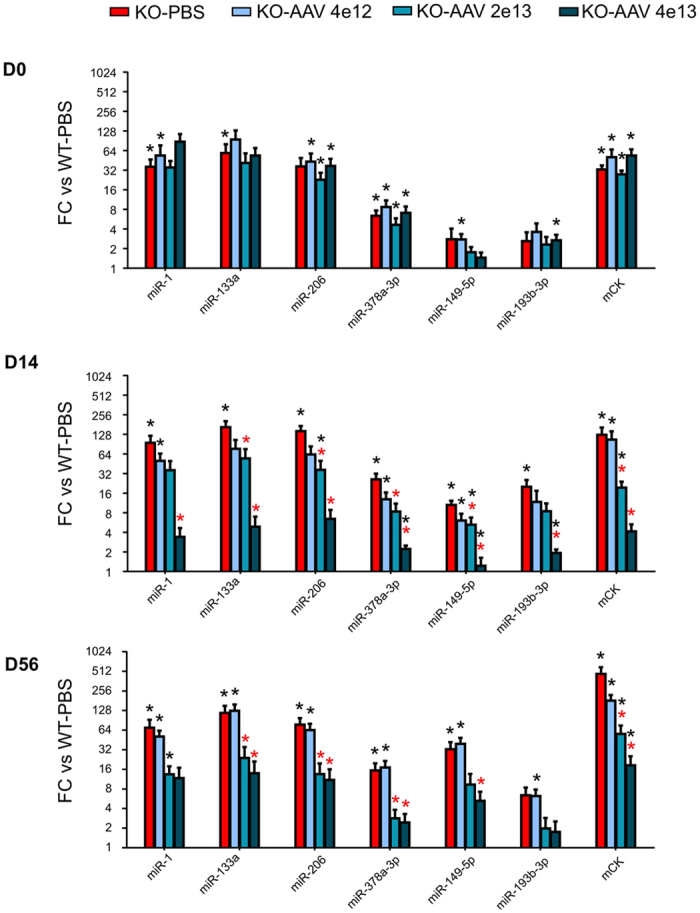
Serum miRNAs and mCK expression in treated mice. MiRNA expression was assessed in the serum of gene-therapy treated KO-*Sgca* dystrophic and healthy control mice, before injection at day zero (D0, upper panel), and after gene therapy by the indicated vector dose, at 14 and 56 days post injection (D14, and D56, middle and lower panels), miRNAs and mCK expressions are presented as fold change (FC) relative to the control C57Bl/6 healthy mouse (WT-PBS). Red and black asterisks designate significant differences compared to untreated KO-*Sgca* (KO-PBS) and to healthy WT control (WT-PBS) mice, respectively. All FC and p values are shown in Supplemental Tables ST4 (D0), ST5 (D14) and ST6 (D56). Note that at D0 (upper panel) all mice are as yet untreated, and the variations in miRNAs FC and p values between viral-dose groups reflect experimental variations.

We evaluated the expression of the dystromiRs (miR-1, miR-133a, miR-206), as well as miR-378a-3p, miR-149-5p and miR-193b-3p in the serum of the treated mice. These 3 last miRs were previously identified as upregulated in the serum of mouse models for the high-regenerative myopathies^25^. Expression of the miRNAs was quantified at three time points, initially before injection (D0), and then at 14 and 56 days after gene delivery. Consistent with our previous report^25^ and the first part of the study, the serum expression levels of all studied miRNAs were elevated at D0 on KO-*Sgca* compared to control healthy mice. In treated KO-*Sgca* mice, serum miRNAs decreased in a dose-response manner (Fig. 4, days-14, -56 and Supplemental Tables ST5 and ST6). On day 14, the expression of miRNAs became non-significantly different (except for miR-149-5p) compared to healthy controls (WT-PBS) in at least one of the higher doses-treated group (Fig. 4, 14 days and Supplemental Table ST5). Further, on the high dose-treated group, at day 14, expressions of all studied miRNAs were significantly different from the untreated dystrophic group (KO-PBS). Similar results were obtained 56 days post transduction, except that, the intermediate dose results were closer to the high dose (Fig. 4 Day 56 and Supplemental Table ST6). Of note, none of the time point for mCK lost their significant difference with the WT although the level became different from the untreated mice. Taken together, this data show that dysregulation of serum miRNA profile was reduced in the treated mice for all tested miRNAs, in direct relation to increased doses of the recombinant vector, transgene expression, and recovery of muscle function.

**Figure 5:**
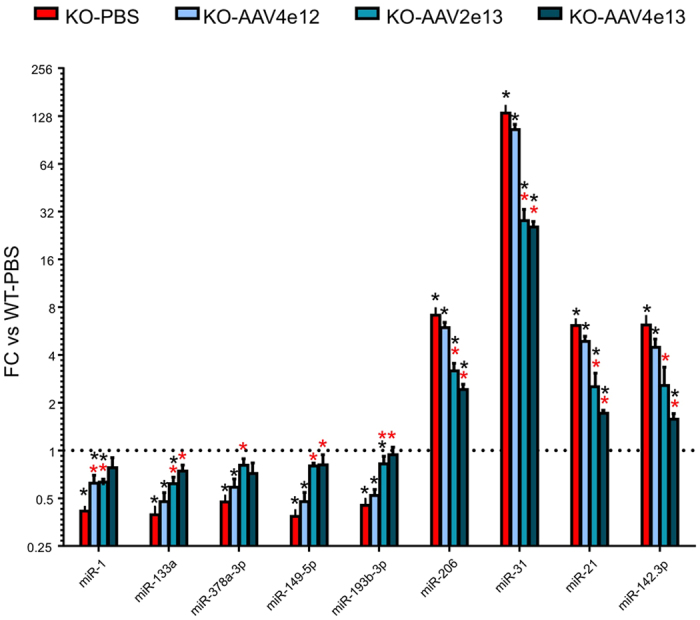
Muscle miRNA expression in treated mice. MiRNA expression was assessed in the Ga muscle of the KO-*Sgca* dystrophic mice, 3 months after transgene delivery, with the indicated viral dose and presented as FC relative to the control C57Bl/6 healthy (WT-PBS) mouse. Red and black asterisks designate significant differences compared respectively to untreated KO-*Sgca* (KO-PBS) and to healthy WT control (WT-PBS) mice. All FC and p values are shown in Supplemental Table ST7.

Expression of miRNAs was evaluated in muscle biopsy at the study endpoint. This investigation included the dystromiRs class (the classical ones miR-1, miR-133, miR-206, and the newly included miR-149-5p, miR-193b-3p and miR-378a-3p), and the three miRNAs known to be dysregulated in dystrophic muscle (miR-21, miR-31, miR-142-3p). Expression of miR-1 and miR-133a, which were identified as downregulated intracellularly in muscles in the 1 and 6 month-old KO-*Sgca* mice, were, as expected, downregulated in the muscle at D0. Similarly, the levels of miR-378a-3p, miR-193b-3p and miR-149-5p were downregulated in the Ga muscle in the dystrophic mice (Fig. 5 and Supplemental Table ST7). Expressions of these miRNAs were upregulated in the muscles of treated mice in correlation to increased doses of the therapeutic viral vector. An inverse pattern was detected for the initially up-regulated miR-206, miR-31, miR-21, and miR-142-3p in untreated dystrophic mice, with a reduction of expression in correlation to viral doses (Fig. 5 and Supplemental Table ST7). Expressions of all miRNAs were significantly different in at least one of the 2 higher vector dose groups, as compared to the untreated dystrophic mice (Fig. 5 and Table ST7). Moreover, with the higher vector dose, the expressions of 5 out of 9 tested miRNAs were no longer significantly dysregulated as compared to the healthy control mice (Fig. 5 and Supplemental Table ST7). Taken together, this data suggest that, at 90 days post transduction, the muscle profiles of all studied miRNAs were showing normalization trends in the treated mice, in correlation to viral dose and recovery of muscle function.

## Discussion

The DAPC structure has an essential role in the mechanical stabilization of the sarcolemma^49^ and is also involved in signal transduction (for a recent review see^50^). Consequently, the pathological mechanism of diseases with loss of DAPC integrity such as DMD or sarcoglycanopathies involved loss of membrane stability and sealing capacity as well as perturbation in signal transduction. Indeed, the dysregulation of miRNAs in DMD patient and animal models^25^,^26^,^27^,^28^,^29^,^30^,^31^,^32^ was proposed to result, in the serum, from increased leakiness of the sarcolemma^30^, and within the muscle tissue, from disruption of a DAPC-dependent signal transduction^51^. However, it remained unclear if and to what extend miRNA dysregulation occurs in the serum in MDs with preserved DAPC and DAPC-dependent signal transduction. In this study, we aimed at investigating the capacity of muscular and circulating miRNAs to be used as generic therapeutic outcome biomarkers in MDs, irrespective of DAPC status. Therefore, we investigated miRNAs expression in serum and muscles of five mouse models, of which three are of disrupted and two of preserved DAPC structure (respectively the HRM and LRM). We observed a tendency for higher miRNA dysregulation in HRM than in LRM, in agreement with the proposition that serum dystromiRs upregulation in MD is subsequent and proportional to the level of damage in the muscle myofibers^30^. The fact that we also observed a similar, albeit with a reduced intensity, perturbation of serum miRNA in the LRM compared to HRM suggests a commonality of signaling pathway dysregulation in all the corresponding diseases that will lead to aberrant miRNA profiles. Specifically, our data support that the upregulation of the dystromiRs miR-1, miR-133 and miR-206 in the serum in MDs is due to their high expression in the muscle and proportional to the level of myofiber degeneration, but irrespective of the primary genetic cause of the distinct pathologies.

Previous studies of mouse models for MD reported that muscle up-regulation of miR-21 is related to tissue fibrosis^34^ and of miR-206 and miR-31 to proliferation of regenerating myoblasts^35^, while the upregulation of miR-142-3p was shown in the present study to originate from infiltrating inflammatory hematopoietic cells. The identification of their dysregulation has been demonstrated in the present study in disease models with both preserved and disrupted DAPC, indicating that the cascades leading to these characteristics of MD are similar across a range of diseases. These data thus support the capacity of miRNAs as generic and versatile biomarkers for a range of MD and variety of pathological aspects in MD.

Interestingly, the variation of miRNAs in the skeletal muscle does not parallel the observation in the serum. The down-regulation of muscle expression of the dystromiRs, miR-1 and miR-133 in DMD animal models and patients has been reported before^30^,^31^,^51^,^52^. This observation is in sharp contrast with the muscle upregulation of miR-31 and miR-206. Of note, miR-31 and miR-206 are expressed highly in proliferating myoblasts and in newly formed myotubes, and thus are upregulated in the dystrophic muscle as a reflection of the regenerative process of MD. In contrast, miR-1 and miR-133 are expressed almost exclusively in mature myofibers. The reduced expression of these two miRNAs might result directly from pathological transcriptional changes in the dystrophic myofiber, or alternatively, from the replacement in MD of the contractile tissue by adipogenic and connective tissues. The observation of miRNA downregulated in dystrophic muscle and upregulated in the serum is extended in the present study to the KO-Sgca mouse model and to the miRNAs miR-378a-3p, miR-193b-3p, and miR-149-5p. The reasons for the dysregulation of miR-378a-3p, miR-193b-3p, and miR-149-5p in MDs are yet unknown. The partial normalization of miR-378a-3p, miR-193b-3p, and miR-149-5p in treated mice supports the idea that this dysregulation is related to pathological changes. It is noteworthy that this pattern of downregulation in dystrophic muscle and upregulation in the serum in MD, is in common with the dystromiRs miR-1 and miR-133 (but not miR-206), which suggest possible similar operating pathological mechanisms.

The aim of the second part of this study was the evaluation of the possibility of monitoring the therapeutic outcome of an experimental approach in MD using miRNAs. For this aim, it was convenient to focus on an established, highly defined, therapeutic model. The α-sarcoglycan mouse is an attractive model, since it has been studied in detail in a number of early studies, and because it presents a well-documented dose response therapeutic benefit^21^,^33^. Analysis of the results of this setup was initially made by standard methodologies, including the expression of the transgene at the RNA and protein levels, muscle histology, and assessment of muscle function. Collectively, these analyses confirmed a dose response in the treated mice, and supported that our system is suitable for the assessment of miRNAs as therapeutic monitoring biomarkers. Importantly, dysregulation of all tested miRNAs, both up- and downregulated in serum and muscles, was reduced as early as 14 days after treatment, in a dose-dependent manner.

Many investigations of circulating miRNAs in MD have identified differentially expressed miRNAs between the disease and control groups. Based on these studies, it could be assumed that miRNA profiling might be used in MD mainly for disease diagnosis. We and others have shown, however, that many of these miRNAs are dysregulated, in common, in distinct MDs, and are therefore of a limited utility for precise diagnosis. In contrast, in therapeutic monitoring, profiling with commonly dysregulated miRNAs might be beneficial, because highly defined biomarkers can be comparatively used in distinct but related diseases that share common secondary manifestations. Indeed, we have demonstrated in the present study a disease-monitoring potential of dysregulated miRNAs in MD. A summary table of the observations that were made in the present and our previous^25^,^32^ studies, concerning the choice of miRNAs and mRNA that might be used for therapeutic monitoring in mouse models for MD, is presented in Table 1. We are proposing thus that miRNA biomarkers in MD are better suited for disease monitoring than for disease diagnosis.

As mentioned already, this study supports that distinct dysregulated miRNAs are potential biomarkers for distinct pathological changes. However, direct causative relations and quantitative correlations between individual miRNAs and specific pathological features were beyond the scope and have not been addressed in the present study. A definitive approval of a specific miRNA for the monitoring of specific pathological aspect will require more detailed characterization of cause and effect relation, as well as detailed quantitative investigation of their correlations. This might be a subject of future investigations, prior to approval of miRNA as a biomarker. The hope, however, is that by profiling circulating miRNAs it will be possible to obtain pertinent information on a vast array of pathological processes, to be monitored individually during medical treatment in MD.

In summary, our data support that circulating miRNAs are generic and versatile biomarkers for therapeutic monitoring in MD. These biomarkers are (i) generic, because amenable for a wide variety of MDs, (ii) versatile, because specific miRNAs are responsive and specific for variety of pathological aspects in MD, and (iii) are useful therapeutic monitoring biomarkers, because their expression is correlating with phenotypical changes in treated mouse model.

## Materials and Methods

### Animal models

Animal experimentations were conducted in accordance with the European guidelines for the protection of vertebrate animals used for experimental purposes (Directive 2010/63/EU of 22 September 2010) and were approved by the ethical committee of Genethon under the number CE 12–034. The following mouse strains were used: C57BL/6 control strain, *mdx*cv4 and four knockout (KO) strains named KO-*Capn3* (model for LGMD2A^41^), KO-*Dysf* (model for LGMD2B^40^), KO-*Sgcg* (model for LGMD2C^38^) and KO-*Sgca* (model for LGMD2D^37^).

### miRNA quantification in serum and muscle tissues

A specific miRNA extraction was performed for serum samples (around 100 μL) using the mirVana^TM^ PARIS^TM^ kit (ThermoFisher). RNA was first eluted in 50 μl RNase-free water and concentrated after NaOAc precipitation. The RNA pellet was finally resuspended in 10 μl RNase free water. For muscle samples, total RNA extraction was performed from frozen sections corresponding to approximately 1 mm thick of muscle transverse sections from *Gastrocnemius Anterior* (Ga) by the Trizol method (Invitrogen). RNA was eluted in 20 μl RNase-free water and treated with Free DNA kit (Ambion) to remove residual DNA. Total RNA extracted for each sample was quantified by using a Nanodrop spectrophotometer (ND8000 Labtech, Wilmington Delaware).

Quantification of miRNAs was performed using Exiqon miRCURY LNA™ Universal RT microRNA PCR. Total RNA (20 ng) was converted into poly-A primed universal cDNA and microRNAs were quantified in duplicate for each sample with miRNA-specific LNA primers on the LightCycler480 (Roche) following manufacturer’s guidelines. Quantification cycle (Cq) values were calculated with the LightCycler® 480 SW 1.5.1 using 2nd Derivative Max method. RT-qPCR results, expressed as raw Cq were normalized to the miRNAs identified as the most stable, miR-16 for individual assays in serum, and miR-93 in muscle samples. The relative expression was calculated using the 2^−ΔCt^ method.

### Quantification of the transgene and endogenous transcripts in muscle

For quantification of the mRNA expression, one μg of RNA was reverse-transcribed using the SuperScript II first strand synthesis kit (Invitrogen) and a mixture of random oligonucleotides and oligo-dT. Real-time PCR was performed using LightCycler480 (Roche) with 0.2 μM of each primer and 0.1 μM of the probe according to the protocol Absolute QPCR Rox Mix (ABgene). The primer pairs and Taqman probes used for the human α-sarcoglycan amplification were: 920hasarco. F:5′-TGCTGGCCTATGTCATGTGC-3′, 991hasarco. R:5′-TCTGGATGTCGGAGGTAGCC-3′, and 946hasarco. P:5′-CGGGAGGGAAGGCTGAAGAGAGACC-3′. The ubiquitous acidic ribosomal phosphoprotein (P0) was used to normalize the data across samples. The primer pairs and Taqman probe used for P0 amplification were: m181P0.F (5′-CTCCAAGCAGATGCAGCAGA-3′), m267P0.R (5′-ACCATGATGCGCAAGGCCAT-3′) and m225P0.P (5′-CCGTGGTGCTGATGGGCAAGAA-3′). The primer pairs and Taqman probes for the muscle endogenous transcripts were Mm01329494_m1 for the mouse myh8; Mm00434455_m1 for the mouse CD11b/Itgam and Mm00711678_m1 for the mouse COL6A3 transcripts (Thermo Fisher Scientific). Each experiment was performed in duplicate.

### AAV-mediated gene transfer

Recombinant adeno-associated virus 8 (rAAV2/8) vector was used to restore α-sarcoglycan expression in KO-Sgca mice. The production of rAAV was performed by dual infection of Sf9 cells with baculoviruses harbouring cDNA for Sgca under expression regulation by desmin promoter and miR-142-3p^36^, and the AAV rep2/cap8 genes. The purification was performed on immuno-affinity AVB sepharose medium (GE Healthcare) according to Smith *et al*.^53^. Viral genomes were quantified by a TaqMan real-time PCR assay using primers and probes for the inverted terminal repeat region (ITR) of the AAV vector genome {Rohr, 2002 #50}. The primer pairs and TaqMan probes used for ITR amplification were: 1AAV65/Fwd: 5′-CTCCATCACTAGGGGTTCCTTGTA-3′; 64AAV65/rev: 5′-TGGCTACGTAGATAAGTAGCATGGC-3′; and AAV65MGB/taq: 5′-GTTAATGATTAACCC-3′. Mice were injected intravenously into the tail vein with a standard volume of 500 μl of either PBS or increasing doses of rAAV [4e12, 2e13, and 4e13 viral genome (vg/kg)].

### Histological and immunohistochemistry analyses

At the end of the protocol, muscles were removed, weighed and quickly frozen in liquid nitrogen-cooled isopentane. Transversal cryosections (8 μm) were prepared from frozen right and left gastrocnemius muscles. Sections were processed for hematoxylin phloxine saffron (HPS) and red Sirius histological staining.

For colorimetric immunodetection of α-sarcoglycan, unfixed transverse cryosections were rehydrated with PBS for 5 min and then incubated with H2O2 to inhibit endogenous peroxydases 20 min at room temperature (RT). After washing with PBS, sections were blocked with PBS/10% goat serum for 30 min and then incubated with 1/1000 dilution of a rabbit polyclonal primary antibody directed against amino acids 366–379 of the human α-sarcoglycan sequence (AC-ahSarco57, Eurogentec^33^) 1 to 2 h at RT. After washing with PBS, sections were incubated with secondary antibody conjugated with horse radish peroxidase (HRP) diluted 1/200 for 1 h at RT. Sections were washed 3 times with PBS and then incubated with diluted diaminobenzidine (DAB; DAKO) for 2–5 min. Then, sections were successively treated with ethanol (5 min), twice in xylene (5 min), mounted with Eukkit (Labonord, France) and visualized on a Nikon microscope.

Immunofluorescent staining was performed to evaluate the level of inflammation (CD11b) and the regenerative status (MHCd) of the muscle. Unfixed transverse cryosections were blocked with PBS/20% FCS for 1 h and then incubated with the primary antibodies against the mouse anti-mouse MHCd (NCL-MHCd, Leica Biosystems, dilution 1:40) and the rat anti-mouse CD11b (Clone M1/70, BD Pharmingen, dilution 1:40) overnight at Room Temperature. After washing with PBS 1x, sections were incubated with a goat anti-rat or goat anti-mouse secondary antibody conjugated with Alexa594 dye diluted 1/600 (ThermoFisher) for 1 h at RT. Sections were mounted with Fluoromount-G and DAPI (SouthernBiotech, USA), and visualized on a fluorescent microscope (Axioplan, Zeiss).

### Evaluation of muscle force

Three months after AAV injection, mouse muscle force was evaluated by the whole body tension (WBT) method or “escape test”^54^ with some modifications. A mouse with a thread attached to one end of its tail and another to a tension transducer was placed on a platform facing the entrance of 30 cm long tube. In response to gentle pinching of the tail, mice tried to escape within the tube thus raising a short peak of force (forward pulling tension, FPT) that was recorded. Five FTP were recorded for each mouse. The body weight of each mouse was measured, and the WBT5 were obtained by dividing the average of the 5 FPTs by the body weight.

### Western blot analysis

Muscle tissues were mechanically homogenized in RIPA lysis buffer (Life technologies), complemented with Complete protease inhibitor cocktail EDTA-free (Roche). Protein samples were separated by 4–12% Bis-Tris gel polyacrylamide gradient NuPAGE gels (Invitrogen) then transferred to nitrocellulose membrane with iBlot® Dry Blot System. After blotting, membranes were probed with antibodies against α-sarcoglycan (mouse monoclonal, Leica NCL-a-SARC, dilution 1:100) and GAPDH (Rabbit polyclonal, SantaCruz, dilution 1:500). Finally, membranes were incubated with IRDye® for revelation by the infrared-scanner Odyssey (LI-COR Biosciences, Lincoln, Nebraska, USA).

### Statistical analyses

Statistical analyses were performed using the GraphPad Prism version 6.04. Data are expressed as mean ± SEM. For comparisons between means, homogeneity of variances was first assessed by Fisher-Snedecor’s test and the appropriate Student’s t-test (two-tailed) was applied. A statistical significance was established at P < 0.05.

## Additional Information

**How to cite this article**: Israeli, D. *et al*. Circulating miRNAs are generic and versatile therapeutic monitoring biomarkers in muscular dystrophies. *Sci. Rep.* **6**, 28097; doi: 10.1038/srep28097 (2016).

## Supplementary Material

Supplementary Information

## Figures and Tables

**Table 1:** A user guide for the choice of miRNA biomarkers and mRNA indicators for therapeutic outcome monitoring in mouse models for muscular dystrophy.

Transcript group	miR-name mRNA name	Dysregulation in mouse models for MD		Pathological indication	Therapeutic response in the KO-*Sgca* mouse model	
		Serum	muscle		Serum	Muscle
DystromiRs	miR-1	Up	Down	Damaged myofibers	Down	Up
	miR-133a	Up	Down		Down	Up
	miR-206	Up	Up		Down	Down
miR indicators of other pathological changes in MD	miR-21	Up	Up	Muscle fibrosis	Down	Down
	miR-31	Varied	Up	MPC proliferation	Varied	Down
	miR-142-3p	Up	Up	Inflammatory infiltration	Down	Down
miR indicators of yet unidentified pathological changes	miR-149-5p	Up	Down	Unknown	Down	Up
	miR-193b-3p	Up	Down		Down	Up
	miR-378a-3p	Up	Down		Down	Up
mRNA indicators	Myh8		Up	Myofiber regeneration		Down
	CD11b		Up	Inflammatory infiltration		Down
	Col6a3		Up	Fibrosis		Down

MD: Muscular Dystrophy. MPC: Muscle precursor cells. Myh8: Myosin heavy chain 8 (neonatal). Col6a3: Collagen 6a3.

**Table 2:** Principal features of mouse strains and dystrophic models.

**Principal features of mouse strains and models**					
Pathology	Mutated gene	Mouse model	Principal affected tissue	Skeletal muscle pathological features	Ref.
α-sarcoglycanopathy, limb-girdle muscular dystrophy type 2D, LGMD2D	α-sarcoglycan *Sgca*	α-sarcoglycan-null, backrossed on C57BL/6, (KO-*Sgca*)	Skeletal muscle	Disrupted DAPC, Massive myofiber degeneration, Robust compensatory regeneration (HRM)	37
γ-sarcoglycanopathy, limb-girdle muscular dystrophy type 2C, LGMD2C	γ-sarcoglycan *Sgcg*	γ-sarcoglycan-null, backrossed on C57BL/6, (KO-*Sgcg*)	Skeletal muscle and heart		38
Duchene muscular dystrophy (DMD)	Dystrophin *Dmd*	Dystrophin-null, 4CV backcrossed on C57BL/6, (mdx)	Skeletal muscle and heart		39
Calpainopathy	Calpain-3 *Capn-3*	Calpain 3-null, backcrossed on C57BL/6, (KO-*Capn3*)	Skeletal muscle	Preserved DAPC, Moderate myofiber degeneration, Low-level compensatory regeneration (LRM)	41
Dysferlinopathy	Dysferlin *Dysf*	Dysferlin-null, backrossed on C57BL/6 (KO-*Dysf*)	Skeletal muscle		40
Healthy control	None	C57BL/6	None		

HRM: high regenerative models LRM: low regenerative models.
