# Supplementary material for: Circulating miRNAs are generic and versatile therapeutic monitoring biomarkers in muscular dystrophies
Source: Sci Rep. 2016 Jun 21;6:28097. doi: 10.1038/srep28097 (PMC4914855; doi:10.1038/srep28097)
Supplement: Supplementary Information [file srep28097-s1.doc]

**Circulating miRNAs are generic and versatile therapeutic monitoring biomarkers in muscular dystrophies**

David Israeli^1,2^*, Jérôme Poupiot^1,2^*, Fatima Amor^1,2^, Karine Charton^1,2^, William Lostal^1,2^, Laurence Jeanson-Leh^2+^, Isabelle Richard^1,2^

Supplementary Information

**Supplemental Figure SF1:**

**MicroiRNA-142-3P is expressed in dystrophic muscle on CD45 +ve mono-nucleated cells (MMNC)**

**Supplemental Figure SF2**

**Longitudinal assessment of serum mCK activity in KO-*Sgca* mice before and after trans-gene delivery**

**Supplemental Figure SF3**

**Relative muscle mass in KO-*Sgca* mouse before and after transgene delivery**

**Supplemental Tables ST1**

**Supplemental Table ST1:** Fold Change and p-value, serum miRNAs, 1 and 6 Month mice.

|  | 1 month old | | | | |  | 6 month old | | | | |
| --- | --- | --- | --- | --- | --- | --- | --- | --- | --- | --- | --- |
| **FC vs WT** | **KO-*Capn3*** | **KO-*Dysf*** | **KO-*Sgcg*** | **KO-Sgca** | **MDX** |  | **KO-*Capn3*** | **KO-*Dysf*** | **KO-*Sgcg*** | **KO-Sgca** | **MDX** |
| miR-1 | 8.5 | 0.3 | 23.0 | 29.1 | 70.5 |  | 38.5 | 60.8 | 624.6 | 15.8 | 322.5 |
| miR-133a | 5.0 | 0.8 | 22.2 | 31.0 | 90.4 |  | 22.0 | 103.0 | 632.4 | 157.6 | 579.6 |
| miR-206 | 7.5 | 0.3 | 20.1 | 18.4 | 31.8 |  | 9.2 | 75.1 | 497.5 | 11.9 | 252.4 |
|  |  |  |  |  |  |  |  |  |  |  |  |
|  | 1 month old | | | | |  | 6 month old | | | | |
| **p value** | **KO-*Capn3*** | **KO-*Dysf*** | **KO-*Sgcg*** | **KO-Sgca** | **MDX** |  | **KO-*Capn3*** | **KO-*Dysf*** | **KO-*Sgcg*** | **KO-Sgca** | **MDX** |
| miR-1 | 3.1E-02 | 3.7E-01 | 3.5E-02 | 1.1E-01 | 8.2E-02 |  | 8.2E-02 | 4.3E-03 | 2.2E-02 | 3.0E-02 | 4.0E-03 |
| miR-133a | 6.7E-03 | 8.2E-01 | 1.7E-02 | 2.1E-02 | 4.7E-02 |  | 4.6E-02 | 1.1E-02 | 2.7E-02 | 1.0E-01 | 2.9E-03 |
| miR-206 | 5.4E-03 | 2.8E-01 | 5.1E-02 | 1.8E-01 | 1.0E-01 |  | 1.1E-01 | 1.6E-03 | 6.2E-02 | 1.7E-02 | 3.2E-02 |

Blue: Down-regulated miRNAs

Red: Upregulated miRNAs

Red-shaded: P values ≤0,05

**Supplemental Tables ST2**

**Supplemental Table ST2:** Fold Change and p-value, muscle miRNAs, 1 and 6 Month mice

|  | 1 month old | | | | |  | 6 month old | | | | |
| --- | --- | --- | --- | --- | --- | --- | --- | --- | --- | --- | --- |
| **FC vs WT** | **KO-*Capn3*** | **KO-*Dysf*** | **KO-*Sgcg*** | **KO-Sgca** | **MDX** |  | **KO-*Capn3*** | **KO-*Dysf*** | **KO-*Sgcg*** | **KO-Sgca** | **MDX** |
| miR-1 | 1,5 | 1,2 | 0.8 | 0.3 | 0.5 |  | 1.5 | 0.8 | 0.7 | 0.3 | 0.5 |
| miR-133a | 1.4 | 1.5 | 0.9 | 0.8 | 0.8 |  | 1.3 | 0.9 | 0.7 | 0.7 | 0.9 |
| miR-206 | 1.6 | 1.8 | 1.7 | 1.5 | 2.8 |  | 1.1 | 4.1 | 5.4 | 8.3 | 8.6 |
| miR-31 | 24.4 | 8.5 | 9.5 | 25.5 | 75.7 |  | 2.5 | 40.3 | 89.8 | 224.3 | 241.5 |
| miR-21 | 1.2 | 2.4 | 1.5 | 4.8 | 7.2 |  | 1.3 | 6.5 | 2.6 | 6.6 | 6.0 |
| miR-142.3p | 1.3 | 1.9 | 1.9 | 6.5 | 5.4 |  | 1.3 | 9.0 | 4.1 | 8.1 | 7.6 |
|  |  |  |  |  |  |  |  |  |  |  |  |
|  | 1 month old | | | | |  | 6 month old | | | | |
| **p value** | **KO-*Capn3*** | **KO-*Dysf*** | **KO-*Sgcg*** | **KO-Sgca** | **MDX** |  | **KO-*Capn3*** | **KO-*Dysf*** | **KO-*Sgcg*** | **KO-Sgca** | **MDX** |
| miR-1 | 2,5E-01 | 6,5E-01 | 6,0E-01 | 8,7E-02 | 3,0E-01 |  | 2,5E-01 | 4,2E-01 | 1,1E-01 | 7,7E-04 | 1,5E-03 |
| miR-133a | 1,2E-01 | 1,8E-01 | 6,6E-01 | 1,1E-01 | 6,2E-01 |  | 1,3E-01 | 6,1E-01 | 1,1E-01 | 2,0E-01 | 3,5E-01 |
| miR-206 | 1,0E-02 | 4,4E-02 | 3,8E-02 | 1,7E-02 | 5,3E-05 |  | 6,1E-01 | 3,8E-02 | 2,8E-02 | 2,8E-03 | 1,1E-03 |
| miR-31 | 4,0E-01 | 1,2E-01 | 8,9E-03 | 7,9E-02 | 1,6E-01 |  | 5,1E-02 | 6,5E-02 | 1,5E-03 | 8,5E-02 | 5,1E-02 |
| miR-21 | 3,9E-01 | 9,9E-02 | 3,0E-01 | 7,0E-02 | 1,7E-01 |  | 1,9E-01 | 2,4E-01 | 5,2E-02 | 4,3E-02 | 1,7E-02 |
| miR-142.3p | 3,4E-01 | 1,6E-02 | 7,0E-02 | 1,5E-02 | 1,9E-01 |  | 4,8E-01 | 2,1E-01 | 7,1E-02 | 6,6E-03 | 8,7E-04 |

Blue: Down-regulated miRNAs

Red: Upregulated miRNAs

Red-shaded: P values ≤0,05

**Supplemental Tables ST3**

**Supplemental Table ST3:** Fold Change and p-value, muscle transcripts, D90 mice

| **Fold Change vs WT** | **Myh8** | **CD11b** | **Col6a3** |
| --- | --- | --- | --- |
| KO_PBS | **134,3** | **3,9** | **5,8** |
| KO_AAV 4e12 | **79,5** | **2,6** | **5** |
| KO_AAV 2e13 | **29,2** | **1,8** | **3,4** |
| KO_AAV 4e13 | **9,6** | **1,5** | **2,3** |
| WT_PBS |  |  |  |
|  |  |  |  |
| **t-test vs KO** | **Myh8** | **CD11b** | **Col6a3** |
| KO_PBS |  |  |  |
| KO_AAV 4e12 | 6,80E-02 | 6,30E-03 | 1,20E-01 |
| KO_AAV 2e13 | 2,40E-03 | 1,30E-03 | 5,20E-02 |
| KO_AAV 4e13 | 3,90E-03 | 1,00E-03 | 4,30E-04 |
| WT_PBS | 3,20E-03 | 5,30E-05 | 3,70E-05 |
|  |  |  |  |
| **t-test vs WT** | **Myh8** | **CD11b** | **Col6a3** |
| KO_PBS | 3,20E-03 | 5,30E-05 | 3,70E-05 |
| KO_AAV 4e12 | 6,60E-03 | 7,90E-04 | 2,20E-04 |
| KO_AAV 2e13 | 7,30E-02 | 9,20E-02 | 4,70E-02 |
| KO_AAV 4e13 | 4,00E-02 | 2,60E-01 | 1,10E-01 |
| WT_PBS |  |  |  |

Red: Upregulated miRNAs

Red-shaded: P values ≤0,05

**Supplemental Tables ST4**

**Supplemental Table ST4:** Fold Change and p-value, serum miRNAs, D0 mice

| **Fold Change vs WT** | **miR-1** | **miR-133a** | **miR-206** | **miR-378a-3p** | **miR-149-5p** | **miR-193b-3p** | **CK** |
| --- | --- | --- | --- | --- | --- | --- | --- |
| KO_PBS | 36.1 | 58.5 | 36.4 | 6.3 | 2.8 | 2.6 | 32.7 |
| KO_AAV 4e12 | 53.7 | 94.7 | 43.3 | 8.7 | 2.8 | 3.6 | 50.5 |
| KO_AAV 2e13 | 35.1 | 41.2 | 22.7 | 4.6 | 1.8 | 2.3 | 27.6 |
| KO_AAV 4e13 | 87.5 | 53.5 | 37.0 | 7.0 | 1.5 | 2.7 | 53.6 |
| WT_PBS |  |  |  |  |  |  |  |
|  |  |  |  |  |  |  |  |
| **t-test vs KO** | **miR-1** | **miR-133a** | **miR-206** | **miR-378a-3p** | **miR-149-5p** | **miR-193b-3p** | **CK** |
| KO_PBS |  |  |  |  |  |  |  |
| KO_AAV 4e12 | 5.1E-01 | 4.1E-01 | 7.3E-01 | 4.1E-01 | 9.9E-01 | 5.5E-01 | 3.4E-01 |
| KO_AAV 2e13 | 9.4E-01 | 5.5E-01 | 3.7E-01 | 3.7E-01 | 4.8E-01 | 8.1E-01 | 4.4E-01 |
| KO_AAV 4e13 | 9.9E-02 | 8.7E-01 | 9.7E-01 | 7.5E-01 | 3.6E-01 | 9.4E-01 | 1.4E-01 |
| WT_PBS |  |  |  |  |  |  |  |
|  |  |  |  |  |  |  |  |
| **t-test vs WT** | **miR-1** | **miR-133a** | **miR-206** | **miR-378a-3p** | **miR-149-5p** | **miR-193b-3p** | **CK** |
| KO_PBS | 2.9E-02 | 5.7E-02 | 5.1E-02 | 1.5E-02 | 2.3E-01 | 1.8E-01 | 2.8E-03 |
| KO_AAV 4e12 | 8.5E-02 | 5.8E-02 | 4.3E-02 | 2.9E-02 | 3.5E-02 | 1.1E-01 | 3.6E-02 |
| KO_AAV 2e13 | 2.1E-02 | 7.6E-02 | 2.7E-02 | 3.9E-02 | 8.0E-02 | 1.4E-01 | 2.4E-03 |
| KO_AAV 4e13 | 5.2E-02 | 5.0E-02 | 4.3E-02 | 4.3E-02 | 2.0E-01 | 2.9E-02 | 2.7E-02 |
| WT_PBS |  |  |  |  |  |  |  |

Red: Upregulated miRNAs

Red-shaded: P values ≤0,05

**Supplemental Tables ST5**

**Supplemental Table ST5:** Fold Change and p-value, serum miRNAs, D14 mice

|  |  |  |  |  |  |  |  |
| --- | --- | --- | --- | --- | --- | --- | --- |
| **Fold Change vs WT** | **miR-1** | **miR-133a** | **miR-206** | **miR-378a-3p** | **miR-149-5p** | **miR-193b-3p** | **CK** |
| KO_PBS | 94.6 | 164.7 | 143.3 | 26.1 | 10.5 | 19.9 | 126.0 |
| KO_AAV 4e12 | 49.9 | 76.7 | 62.9 | 12.9 | 6.1 | 11.7 | 105.9 |
| KO_AAV 2e13 | 35.9 | 54.6 | 36.4 | 8.3 | 5.2 | 8.4 | 19.4 |
| KO_AAV 4e13 | 3.4 | 4.9 | 6.4 | 2.2 | 1.2 | 1.9 | 4.1 |
| WT_PBS |  |  |  |  |  |  |  |
|  |  |  |  |  |  |  |  |
| **t-test vs KO** | **miR-1** | **miR-133a** | **miR-206** | **miR-378a-3p** | **miR-149-5p** | **miR-193b-3p** | **CK** |
| KO_PBS |  |  |  |  |  |  |  |
| KO_AAV 4e12 | 1.8E-01 | 1.0E-01 | 5.2E-02 | 8.2E-02 | 8.9E-02 | 3.2E-01 | 7.1E-01 |
| KO_AAV 2e13 | 8.7E-02 | 3.6E-02 | 9.7E-03 | 2.2E-02 | 4.2E-02 | 9.5E-02 | 4.5E-02 |
| KO_AAV 4e13 | 2.7E-02 | 1.4E-02 | 8.6E-03 | 1.3E-02 | 3.7E-03 | 3.0E-02 | 3.1E-02 |
| WT_PBS | 2.5E-02 | 1.3E-02 | 7.7E-03 | 1.1E-02 | 4.0E-03 | 2.5E-02 | 2.8E-02 |
|  |  |  |  |  |  |  |  |
| **t-test vs WT** | **miR-1** | **miR-133a** | **miR-206** | **miR-378a-3p** | **miR-149-5p** | **miR-193b-3p** | **CK** |
| KO_PBS | 2.5E-02 | 1.3E-02 | 7.7E-03 | 1.1E-02 | 4.0E-03 | 2.5E-02 | 2.8E-02 |
| KO_AAV 4e12 | 3.1E-02 | 5.4E-02 | 3.9E-02 | 2.7E-02 | 3.6E-02 | 1.3E-01 | 4.5E-02 |
| KO_AAV 2e13 | 6.5E-02 | 6.7E-02 | 5.8E-02 | 5.1E-02 | 4.7E-02 | 5.4E-02 | 1.5E-02 |
| KO_AAV 4e13 | 1.0E-01 | 1.6E-01 | 1.1E-01 | 4.6E-03 | 5.6E-01 | 7.4E-03 | 7.3E-02 |
| WT_PBS |  |  |  |  |  |  |  |

Red: Upregulated miRNAs

Red-shaded: P values ≤0,05

**Supplemental Tables ST6**

**Supplemental Table ST6:** Fold Change and p-value, serum miRNAs, D56 mice

| **Fold Change vs WT** | **miR-1** | **miR-133a** | **miR-206** | **miR-378a-3p** | **miR-149-5p** | **miR-193b-3p** | **mCK** |
| --- | --- | --- | --- | --- | --- | --- | --- |
| KO_PBS | 69.6 | 117.2 | 77.8 | 15.3 | 32.6 | 6.4 | 466.5 |
| KO_AAV 4e12 | 51.1 | 126.9 | 64.4 | 17.1 | 39.3 | 6.2 | 181.8 |
| KO_AAV 2e13 | 13.5 | 24.0 | 13.5 | 2.8 | 9.4 | 2.0 | 56.1 |
| KO_AAV 4e13 | 11.8 | 13.9 | 11.0 | 2.5 | 5.3 | 1.7 | 18.5 |
| WT_PBS |  |  |  |  |  |  |  |
|  |  |  |  |  |  |  |  |
| **t-test vs KO** | **miR-1** | **miR-133a** | **miR-206** | **miR-378a-3p** | **miR-149-5p** | **miR-193b-3p** | **CK** |
| KO_PBS |  |  |  |  |  |  |  |
| KO_AAV 4e12 | 4.9E-01 | 8.4E-01 | 6.1E-01 | 7.8E-01 | 6.2E-01 | 9.4E-01 | 8.3E-02 |
| KO_AAV 2e13 | 6.9E-02 | 4.8E-02 | 3.0E-02 | 4.7E-02 | 5.1E-02 | 7.7E-02 | 2.9E-02 |
| KO_AAV 4e13 | 6.3E-02 | 3.6E-02 | 2.7E-02 | 4.3E-02 | 3.9E-02 | 8.9E-02 | 2.2E-02 |
| WT_PBS |  |  |  |  |  |  |  |
|  |  |  |  |  |  |  |  |
| **t-test vs WT** | **miR-1** | **miR-133a** | **miR-206** | **miR-378a-3p** | **miR-149-5p** | **miR-193b-3p** | **mCK** |
| KO_PBS | 4.0E-02 | 2.6E-02 | 1.8E-02 | 3.2E-02 | 2.6E-02 | 5.3E-02 | 2.0E-02 |
| KO_AAV 4e12 | 1.3E-02 | 1.6E-02 | 1.6E-02 | 2.1E-02 | 1.5E-02 | 3.1E-02 | 1.1E-02 |
| KO_AAV 2e13 | 4.6E-02 | 1.1E-01 | 1.1E-01 | 1.4E-01 | 1.2E-01 | 3.4E-01 | 4.6E-02 |
| KO_AAV 4e13 | 1.3E-01 | 1.7E-01 | 1.4E-01 | 1.9E-01 | 1.2E-01 | 4.2E-01 | 8.3E-02 |
| WT_PBS |  |  |  |  |  |  |  |

Red: Upregulated miRNAs and mCK

Red-shaded: P values ≤0,05

**Supplemental Tables ST7**

**Supplemental Table ST7:** Fold Change and p-value, muscle miRNAs, D90 mice

| **Fold Change** | **miR-1** | **miR-133a** | **miR-378a-3p** | **miR-149-5p** | **miR-193b-3p** | **miR-206** | **miR-31** | **miR-21** | **miR-142-3p** |
| --- | --- | --- | --- | --- | --- | --- | --- | --- | --- |
| KO_PBS | 0.41 | 0.40 | 0.59 | 0.45 | 0.38 | 7.13 | 133.79 | 6.12 | 6.16 |
| KO AAV 1e11 | 0.62 | 0.48 | 0.59 | 0.52 | 0.48 | 5.94 | 105.23 | 4.87 | 4.46 |
| KO AAV 5e11 | 0.63 | 0.62 | 0.80 | 0.82 | 0.80 | 3.17 | 28.06 | 2.52 | 2.57 |
| KO AAV 1e12 | 0.78 | 0.74 | 0.71 | 0.94 | 0.81 | 2.42 | 25.61 | 1.72 | 1.57 |
| WT_PBS |  |  |  |  |  |  |  |  |  |
|  |  |  |  |  |  |  |  |  |  |
| **P value vs KO** | **miR-1** | **miR-133a** | **miR-378a-3p** | **miR-149-5p** | **miR-193b-3p** | **miR-206** | **miR-31** | **miR-21** | **miR-142-3p** |
| KO_PBS |  |  |  |  |  |  |  |  |  |
| KO AAV 1e11 | 4,9E-02 | 3,5E-01 | 2,4E-01 | 2,9E-01 | 2,7E-01 | 2,1E-01 | 1,2E-01 | 1,1E-01 | 1,4E-01 |
| KO AAV 5e11 | 9,2E-03 | 2,0E-02 | 7,6E-03 | 7,1E-03 | 3,0E-05 | 1,2E-03 | 9,9E-04 | 2,1E-03 | 1,6E-02 |
| KO AAV 1e12 | 5,4E-02 | 3,3E-03 | 8,0E-02 | 2,7E-03 | 4,2E-02 | 2,1E-03 | 1,5E-03 | 1,6E-03 | 5,8E-03 |
| WT_PBS | 6,6E-04 | 9,8E-06 | 2,8E-05 | 2,0E-05 | 3,1E-06 | 6,3E-05 | 7,7E-04 | 8,2E-04 | 3,9E-03 |
|  |  |  |  |  |  |  |  |  |  |
| **P value vs WT** | **miR-1** | **miR-133a** | **miR-378a-3p** | **miR-149-5p** | **miR-193b-3p** | **miR-206** | **miR-31** | **miR-21** | **miR-142-3p** |
| KO_PBS | 6,6E-04 | 9,8E-06 | 2,8E-05 | 2,0E-05 | 3,1E-06 | 6,3E-05 | 7,7E-04 | 8,2E-04 | 3,9E-03 |
| KO AAV 1e11 | 7,2E-03 | 1,5E-04 | 1,3E-03 | 6,9E-05 | 1,7E-04 | 2,9E-05 | 2,2E-04 | 2,8E-04 | 3,2E-03 |
| KO AAV 5e11 | 4,1E-03 | 7,2E-04 | 6,4E-02 | 1,3E-01 | 4,4E-03 | 2,6E-03 | 5,6E-03 | 4,7E-02 | 1,2E-01 |
| KO AAV 1e12 | 1,5E-01 | 8,9E-03 | 9,3E-02 | 6,0E-01 | 2,4E-01 | 1,4E-02 | 1,2E-03 | 8,4E-04 | 1,1E-02 |
| WT_PBS |  |  |  |  |  |  |  |  |  |

Blue: Down-regulated miRNAs

Red: Upregulated miRNAs

Red-shaded: P values ≤0,05

**Supplemental Figures legends**

**Supplemental Figure SF1:**

MiRNAs expression was investigated in the dystrophic KO-*SGCA* and the healthy control C57Bl/6 mice. MiR-1 and miR-142-3p were quantified in the tibialis anterior (TA), and in the hematopoietic fraction (CD45+) of the muscle-derived mono-nucleated cells (MMNC). Detection of miRNAs in the spleen was used as positive and negative control respectively for miR-142-3p and miR-1.

**Supplemental Figure SF2:**

Longitudinal assessment of serum mCK enzymatic activity on the indicated days after transgene delivery and viral vector dose. P values are indicated in the table. Red-shaded P value ≤0,05

**Supplemental Figure SF3:**

Muscle weight relative to control mouse, expressed in fold change, (Bi) biceps, (TA) Tibialis Anterior, (Qua) Quadriceps, (Ga) Gastrocnemius, (Total Body) total body weight. Red and black asterisks indicate the significant difference compared to untreated KO-Sgca (KO-PBS) and to healthy control mouse (WT-PBS), respectively.

**Supplemental Materials and Methods**

**Supplemental m&m Figure SF1**

**MiRNA expression in the TA muscle and MMNC:** Tibialis anterior muscle form KO-Sgca mice were dissociated enzymatically with collagenase A (Roche) 1% in DMEM medium (ThermoFisher Scientific) for 30 minutes at 37°C, placed in the Thermomixer (Eppendorf) under vigorous shaking. Total muscle mono-nucleated cells (MMNC) were separated from muscle fibers using cell strainer. Hematopoietic MMNC (CD45+ MMNC) were isolated by magnetic-activated cell sorting (MACS) using CD45 microbeads (Miltenyi Biotec). A miRNA profiling of this selected cells was performed using Taqman Low Density Array (TLDA) cards (ThermoFisher Scientific). Total RNA (350 ng) extracted with Trizol method was reverse-transcribed using the Megaplex Primer Pools A and B (mouse version 2) and microRNAs were quantified with TaqMan® Array. MicroRNA Cards A and B (mouse version 2) on the 7900HT Real-Time PCR System (ThermoFisher Scientific) following manufacturer’s guidelines. Quantification cycle (Cq) values were calculated with the SDS software v2.3 using automatic baseline with a threshold fixed at 0.1. The relative expression of miR-1 and miR-142.3p was calculated using the 2-ΔCt method and miR-93 as a normalizer.

**Supplemental m&m Figure SF2**

**Serum mCK quantification:** Blood samples were collected biweekly for 3 months by retro-orbital bleeding and quickly centrifuged 10 minutes at 8000 rpm. Sera were harvested and further centrifuged to completely remove cells contaminants. The sera were finally stored at −80°C until measurement. The quantification of mCK activity was performed using the Vitros DT60 II Chemistry System according to the manufacturer's instructions (Ortho-Clinical Diagnostics).
